# Centrosomal and Non-centrosomal Functions Emerged through Eliminating Centrosomes

**DOI:** 10.1247/csf.20007

**Published:** 2020-04-09

**Authors:** Yutaka Takeda, Kanako Kuroki, Takumi Chinen, Daiju Kitagawa

**Affiliations:** 1 Department of Physiological Chemistry, Graduate School of Pharmaceutical Science, The University of Tokyo, Bunkyo, Tokyo 113-0033, Japan

**Keywords:** centrosome, centrinone, mitotic spindle, bipolarity, NuMA

## Abstract

Centrosomes are highly conserved organelles that act as the major microtubule-organizing center (MTOC) in animal somatic cells. Through their MTOC activity, centrosomes play various roles throughout the cell cycle, such as supporting cell migration in interphase and spindle organization and positioning in mitosis. Various approaches for removing centrosomes from somatic cells have been developed and applied over the past few decades to understand the precise roles of centrosomes. Centrinone, a reversible and selective PLK4 (polo-like kinase 4) inhibitor, has recently emerged as an efficient approach to eliminate centrosomes. In this review, we describe the latest findings on centrosome function that have been revealed using various centrosome-eliminating approaches. In addition, we discuss our recent findings on the mechanism of centrosome-independent spindle bipolarization, discovered through the use of centrinone.

## Centrosome cycle and its function

**Fig. 1:**
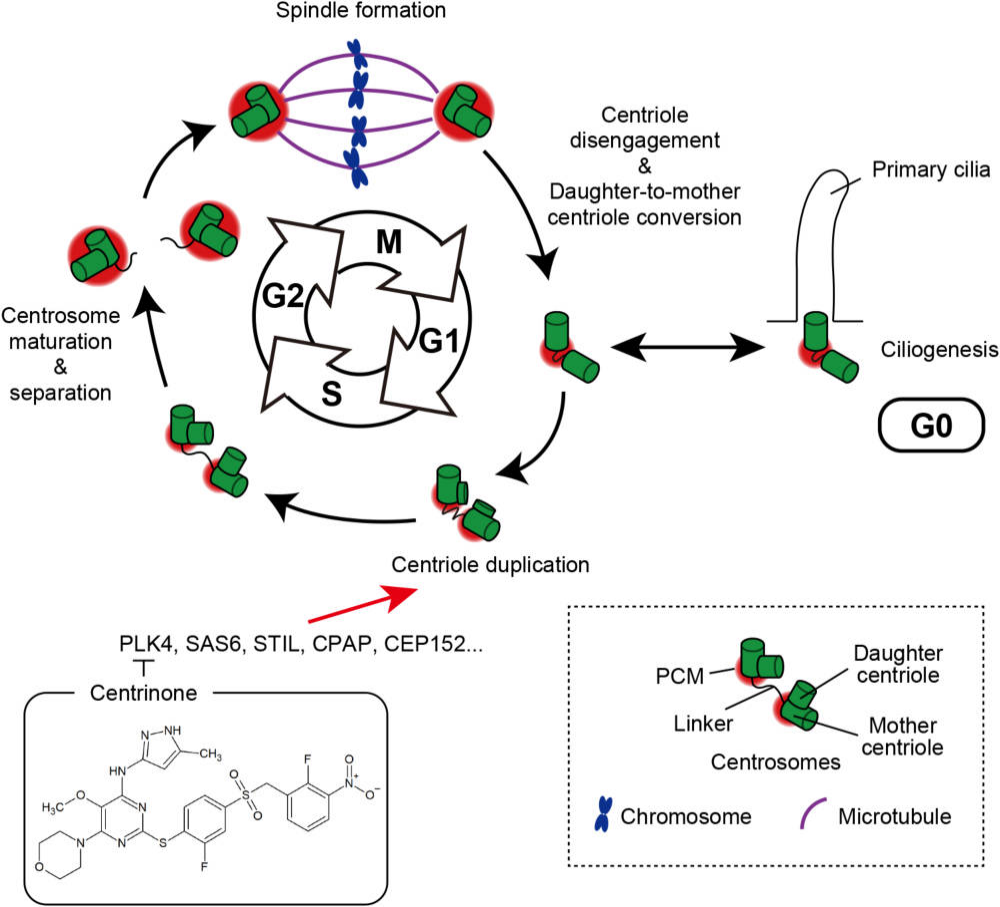
The centrosome cycle. In mitosis, a bipolar spindle is formed and centrosomes function as the poles of the spindle. After cell division, the connection between the mother and daughter centrioles is resolved (centriole disengagement), and the daughter centriole recruits PCM proteins and becomes a mother centriole. When cells stop proliferating and enter G0 phase, centrosomes act as basal bodies for the growth of cilia (ciliogenesis). In S phase, the process of centriole duplication is promoted by several factors (e.g., PLK4, SAS6, STIL, CPAP, and CEP152). Starting at G2 phase, the two centrosomes experience extreme PCM expansions, which allow for stronger MTOC activity (centrosome maturation). At G2/M transition, the two centrosomes are separated from each other and move to the opposite sides of the mitotic cell (centrosome separation), followed by initiation of the next cell division process.

The centrosome is a highly conserved membrane-less organelle, which is composed of one or two centrioles surrounded by pericentriolar material (PCM) (Conduit *et al.*, 2015; Nigg and Holland, 2018; Nigg and Raff, 2009). Centrosomes mainly function as the major microtubule-organizing center (MTOC) in somatic animal cells (Conduit *et al.*, 2015), and undergo multiple events throughout the cell cycle (Fig. 1). Centrioles are duplicated once per cell cycle during S phase (Nigg and Holland, 2018). In this duplication process, only a single daughter centriole is formed next to each mother centriole (Ohta *et al.*, 2014; Rattner and Phillips, 1973). Several factors critical for centriole duplication have been identified, such as PLK4, SAS6, STIL, CPAP, and CEP152 (Bettencourt-Dias *et al.*, 2005; Cizmecioglu *et al.*, 2010; Dammermann *et al.*, 2004; Dzhindzhev *et al.*, 2010; Habedanck *et al.*, 2005; Hatch *et al.*, 2010; Kleylein-Sohn *et al.*, 2007; Leidel *et al.*, 2005; Lin *et al.*, 2013; Stevens *et al.*, 2010; Tang *et al.*, 2011). After centriole duplication, the process of centrosome maturation is initiated in G2 phase with an extreme expansion of PCM, allowing centrosomes to acquire robust MTOC activity (Conduit *et al.*, 2014; Izquierdo *et al.*, 2014; Paweletz *et al.*, 1984). At the G2/M transition, the two centrosomes are separated from each other and move to the opposite sides of the mitotic cell (centrosome separation) (Raaijmakers *et al.*, 2012; Rattner and Berns, 1976; Toso *et al.*, 2009; Waters *et al.*, 1993; Whitehead and Rattner, 1998). During mitosis, centrosomes localize at the mitotic spindle poles (Nigg and Holland, 2018). Following cell division, the connection between the mother and daughter centrioles is resolved (centriole disengagement) (Tsou *et al.*, 2009; Tsou and Stearns, 2006; Vidwans *et al.*, 1999), and the daughter centriole recruits PCM proteins and becomes a mother centriole (daughter-to-mother centriole conversion). After the conversion process, both centrioles obtain the ability to duplicate (Fu *et al.*, 2016; Izquierdo *et al.*, 2014; Tsou *et al.*, 2009; Tsou and Stearns, 2006; Tsuchiya *et al.*, 2016). Overall, centrosomes undergo many processes throughout the cell cycle and their number is strictly regulated in animal somatic cells.

Centrosomes play various crucial roles in animal cells through their MTOC activity. During interphase, centrosomes regulate the microtubule network and govern cell motility (Werner *et al.*, 2017). During mitosis, centrosomes function as the poles of the mitotic spindle to maintain spindle structure and spindle positioning for correct chromosome segregation (Conduit *et al.*, 2015).

## Centrosome-eliminating approaches developed over the past few decades

To investigate the necessity of centrosomes in animal somatic cells for proper mitotic spindle formation and chromosome segregation, several groups have eliminated centrosomes from these cells through different technical approaches. Traditionally, microsurgery or depletion of centriole duplication factors have been used to remove centrosomes (Table I). Khodjakov *et al.* succeeded in selectively removing centrosomes without damaging surrounding structures by laser microsurgery targeting GFP-tagged γ-tubulin (a centrosome marker) in mammalian cells (Khodjakov *et al.*, 2000). They reported that bipolar mitotic spindles were formed without centrosomes. Basto *et al.* created DSas-4 (an ortholog of human CPAP) mutant flies, whose cells do not have centrosomes. They confirmed that centrosomes are not necessary for most aspects of *Drosophila* development (Basto *et al.*, 2006). Hornick *et al.* performed microsurgery with a microneedle to obtain acentrosomal mammalian cells (Hornick *et al.*, 2011). They found that the fidelity of bipolar mitotic spindle assembly is highly compromised in the absence of centrosomes. Sir *et al.* obtained chicken cells lacking centrosomes through knockout of CEP152 or STIL (Sir *et al.*, 2013). These cells showed delays in bipolar spindle formation and high rates of chromosome segregation errors. In summary, the presence of centrosomes in animal somatic cells is important for proper mitotic progression to some extent, but, in many cases centrosomes are not essential. However, the detailed mechanisms of centrosome-independent bipolar spindle formation in somatic cells have not been extensively discussed.

## Emergence of centrinone: a reversible and selective inhibitor of PLK4

All the above methods for the removal of centrosomes require special devices for microsurgery or efficient systems for gene knockdown or knockout. In contrast, centrinone, a reversible and selective PLK4 inhibitor developed in 2015 (Wong *et al.*, 2015), has enabled the easy removal of centrosomes from living animal cells. Wong *et al.* selected VX-680, a pan-Aurora kinase inhibitor which also inhibits PLK4 (Harrington *et al.*, 2004; Sloane *et al.*, 2010), as a template to develop selective PLK4 inhibitors. Through the introduction of a methoxy substituent at the VX-680 C5 position and further optimization, they succeeded in synthesizing centrinone and centrinone-B. Both inhibitors exhibited >1,000-fold selectivity for PLK4 over Aurora kinases (Wong *et al.*, 2015). After treatment with centrinone, they observed cell cycle arrest in G1 phase in normal human cells (RPE-1 cells) and confirmed that the arrest was not due to previously described stress responses to DNA damage, Hippo signaling, or chromosome segregation errors. On the other hand, they noted that cancer cell lines could proliferate in the absence of centrosomes, and the proliferation rates were not correlated with the basal frequency of centrosome amplification observed in those cell lines. These results suggest that numerous cancer cells have abolished the response system that arrests the cell cycle after centrosome loss.

Since the advent of centrinone, other various studies have used this agent (Table II). Meitinger *et al.* performed a genome-wide CRISPR/Cas9 screen in RPE-1 cells and identified a 53BP1-USP28 module which induces G1-phase cell cycle arrest after centrosome loss (Meitinger *et al.*, 2016). Two other groups identified the same pathway by using another method to remove centrosomes from human somatic cells (Fong *et al.*, 2016; Lambrus *et al.*, 2016). Gheiratmand *et al.* performed BioID experiments targeting seven centriolar satellite components while treating cells with centrinone. BioID is a method to screen for protein interactions in living cells using biotin ligase. The ligase fused to the protein of interest biotinylates proximal proteins and enables their isolation and identification (Roux *et al.*, 2012). Using this technique, they revealed that the interactions among centriolar satellite proteins are not affected by centrosome removal (Gheiratmand *et al.*, 2019). Another group performed similar experiments using the knockout of STIL or CEP152 to remove centrosomes, and reached the same conclusion (Quarantotti *et al.*, 2019). Collectively, these results suggest that treatment with centrinone is as effective as other existing methods. The Golgi apparatus also exhibits MTOC activity and localizes around centrosomes (Chabin-Brion *et al.*, 2001; Efimov *et al.*, 2007; Rivero *et al.*, 2009; Ríos *et al.*, 2004). Centrinone has been used in several studies to investigate the relationship and dependency between the two organelles (Guizzunti and Seemann, 2016; Wu *et al.*, 2016). Moreover, Dudka *et al.* produced human cells which have only one centrosome during mitosis through treatment with centrinone (Dudka *et al.*, 2019). They used these cells as a tool for studying k-fiber dynamics in cells with asymmetric bipolar spindles and found that the centrosome regulated k-fiber plus-ends in a HURP-dependent manner. Overall, owing to its ease of use, centrinone has made a huge impact on the latest developments in cell biology. However, a detailed study on the mechanisms of acentrosomal spindle formation using centrinone has not been performed.

## A centrosome-independent mechanism of spindle bipolarity establishment revealed using centrinone

Based on the aforementioned evidence, we used centrinone to investigate the machinery of bipolar spindle formation in acentrosomal human cells. The establishment of a bipolar spindle is crucial for equational chromosome segregation (Prosser and Pelletier, 2017). In somatic cells, spindle bipolarity is achieved through centrosome separation, which occurs ahead of or following nuclear envelope breakdown (NEBD) (Beaudouin *et al.*, 2002; Kaseda *et al.*, 2012; Raaijmakers *et al.*, 2012; Rattner and Berns, 1976; Toso *et al.*, 2009; Waters *et al.*, 1993; Whitehead and Rattner, 1998). During centrosome separation, two centrosomes linked by antiparallel microtubules are pushed away from each other through microtubule crosslinking and the sliding activity of kinesin Eg5 (Bertran *et al.*, 2011; Hata *et al.*, 2019; Kapitein *et al.*, 2005; Kaseda *et al.*, 2012; Smith *et al.*, 2011). During mitosis, these separated centrosomes function as the core of spindle poles and organize microtubules into a bipolar spindle.

On the other hand, meiotic spindles are formed without centrosomes in the oocytes of many species, including flies, frogs, mice, and humans (Calarco-Gillam *et al.*, 1983; Heald *et al.*, 1996; Holubcová *et al.*, 2015; Matthies *et al.*, 1996). In addition, the somatic cells of flies and vertebrates can organize functional bipolar spindles following the removal of centrosomes by lasers, microneedles, or mutations of centriole duplication factors (Basto *et al.*, 2006; Bonaccorsi *et al.*, 2000; Hornick *et al.*, 2011; Khodjakov *et al.*, 2000; Sir *et al.*, 2013). Therefore, it appears that the establishment of spindle bipolarity is not completely dependent on centrosome separation, and an acentrosomal pathway may exist as a compensation mechanism. The *in vitro* reconstitution of meiotic spindles utilizing *Xenopus* egg extracts has been a useful model system for the study of acentrosomal spindle formation (Cross and Powers, 2009). In this system, microtubules are nucleated via chromatin- and Ran-based pathways (Carazo-Salas *et al.*, 1999; Heald *et al.*, 1996; Kalab *et al.*, 1999; Karsenti *et al.*, 1984; Wilde, 1999; Zhang *et al.*, 1999) and are presumably rearranged into a bipolar state in a microtubule- and motor protein-dependent manner (Groen *et al.*, 2008; Heald *et al.*, 1996; Loughlin *et al.*, 2010; Petry *et al.*, 2011). In addition, in mouse oocytes, multiple MTOCs that contain PCM are formed *de novo*, and these MTOC foci are eventually clustered into a bipolar state in an Eg5-dependent manner (Schuh and Ellenberg, 2007). However, other factors involved in acentrosomal spindle bipolarization, besides microtubules, motor proteins, and PCM components have not been extensively investigated thus far.

NuMA (nuclear mitotic apparatus) protein, one of the spindle pole components, directly interacts with microtubules (Du *et al.*, 2002; Haren and Merdes, 2002) and presumptively organizes centrosome-independent microtubule asters (Du *et al.*, 2002; Gaglio *et al.*, 1995; Haren and Merdes, 2002). This protein is necessary for focusing microtubules at spindle poles (spindle pole organization) (Merdes *et al.*, 2000, 1996). In addition, cortical NuMA creates spindle-pulling forces in coordination with the dynein-dynactin complex, placing the spindle at an appropriate position within the cell (Du and Macara, 2004; Okumura *et al.*, 2018). However, the role of NuMA in spindle bipolarization, rather than spindle pole organization and spindle positioning, is not completely understood.

We treated human cells with centrinone to create artificial acentrosomal cells (Chinen *et al.*, 2020). We confirmed that these cells displayed prolonged mitotic duration and increased frequency of chromosome segregation errors, consistent with previous studies using centrinone (Meitinger *et al.*, 2016; Wong *et al.*, 2015). These results suggest that mitotic fidelity is compromised in the absence of centrosomes in human cells as well as other vertebrates (Sir *et al.*, 2013). Subsequently, we investigated the role of NuMA in spindle bipolarization in acentrosomal cells. We found that in acentrosomal cells, shortly after NEBD, NuMA formed several aggregates that organized microtubule asters. Subsequently, these aggregates assembled into two NuMA structures (initial bipolarity establishment), in a manner dependent on microtubules, dynein, and the clustering activity of NuMA. These two structures, located close to each other, organized a radial array of microtubules around them. This radial array of microtubules incorporated Eg5. Eventually, these two NuMA structures were separated to form a bipolar spindle. The separation of the two NuMA structures is dependent on Eg5 motor activity and kinetochore-microtubule attachment. Disruption of Eg5 motor activity or kinetochore-microtubule attachment resulted in fusion of the two NuMA structures after the initial bipolarity was established, leaving cells in a monopolar-like state. Following the depletion of NuMA in acentrosomal cells by auxin-inducible degradation (Natsume *et al.*, 2016; Nishimura *et al.*, 2009; Okumura *et al.*, 2018), cells failed to establish bipolar spindles.

**Fig. 2:**
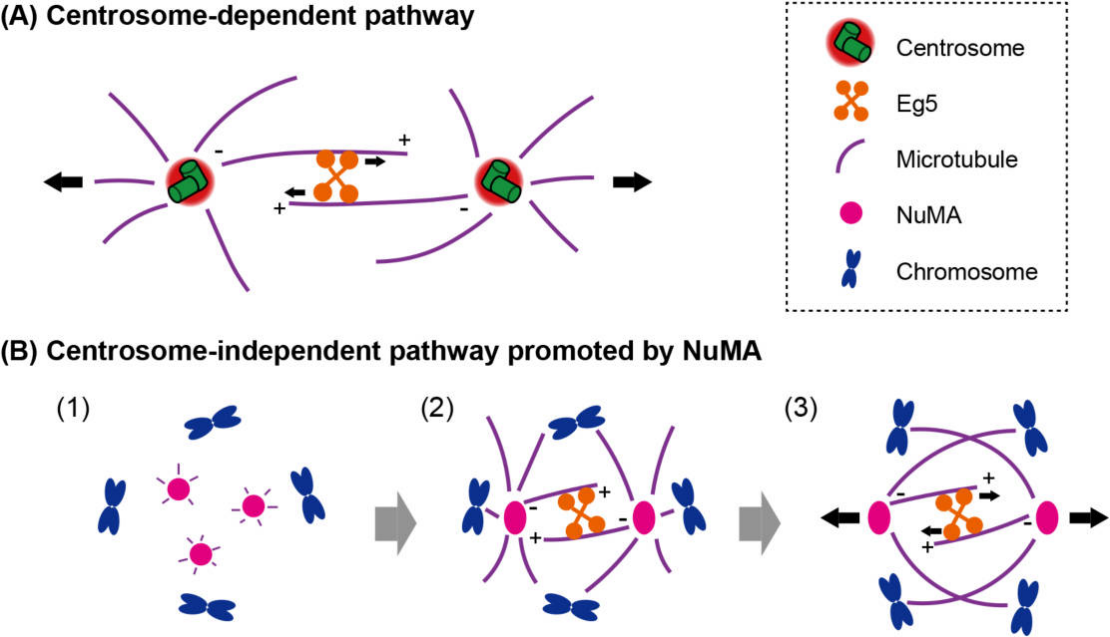
Models for two pathways that promote spindle bipolarization in human cells. (A) The canonical centrosomal pathway. At G2/M transition, the two centrosomes are pushed apart by the plus-end-directed motor activity of kinesin Eg5. (B) The NuMA-mediated pathway, which occurs independently of centrosomes. (1) At the onset of mitosis, NuMA aggregates and organizes microtubule asters. (2) Dynein activity and the clustering activity of NuMA assembles the NuMA aggregates into two poles; subsequently, Eg5 is loaded onto the antiparallel microtubules. (3) Spindle poles are separated through Eg5 motor activity and kinetochore-microtubule attachments.

Importantly, we found that NuMA promoted the initial steps of spindle bipolarization in early mitosis even in cells with centrosomes (Chinen *et al.*, 2020). We observed that the time from NEBD to bipolarity establishment prolonged upon depletion of NuMA. The results of our study suggest that the canonical centrosomal pathway and the NuMA-mediated acentrosomal pathway complementally regulate bipolar spindle assembly in somatic cells, and that the latter becomes predominant in the absence of centrosomes (Fig. 2). Understanding the multiple pathways that ensure robust bipolar spindle formation will assist in the design of anticancer drugs that target spindle assembly (Henriques *et al.*, 2019; Tischer and Gergely, 2019).

## Concluding remarks

Since its development in 2015, centrinone has allowed for great advances in our understanding of centrosome function and the consequences of centrosome loss. Its ease of use has led to the success of numerous large-scale screens that shed light on the interplay between centrosomes and other organelles or cellular systems. Moreover, centrinone has enabled a detailed analysis of the cell division machinery in acentrosomal cells. This analysis assists us in understanding the complemental mechanisms through which the mitotic spindle is regulated by both centrosome-dependent and centrosome-independent pathways.

## Figures and Tables

**Table I:** Examples of studies on acentrosomal somatic cells

Species/cell	Approach	Main finding	Reference
Monkey/CVG-2	Laser microsurgery	A bipolar mitotic spindle is formed in the absence of centrosomes.	Khodjakov *et al.*, 2000
Fly	Mutant of DSas-4	Centrosomes are not necessary for the development of the fly.	Basto *et al.*, 2006
Monkey/BSC-1	Needle microsurgery	The fidelity of the bipolar mitotic spindle is highly compromised in the absence of centrosomes.	Hornick *et al.*, 2011
Chicken/DT40	Knockout of CEP152/STIL	Centrosome loss leads to delays in spindle formation and high rates of chromosomal instability.	Sir *et al.*, 2013

**Table II:** Examples of studies using centrinone and human cells

Cell	Approach	Main finding	Reference
BT549, Calu-6, HCT116, HeLa, MDA-MB-231, RPE-1, U2OS	Centrinone	Centrosome loss leads to G1-phase cell cycle arrest in normal cells, but not in cancer cells.	Wong *et al.*, 2015
SV589 HeLa	Centrinone	The link between the Golgi apparatus and centrosomes must be dissolved to reach metaphase.	Guizzunti and Seemann, 2016
RPE-1	Centrinone	A single Golgi can be maintained in the absence of centrosomes.	Wu *et al.*, 2016
RPE-1	Centrinone CRISPR/Cas9 screen	The 53BP1-USP28 module induces G1-phase cell cycle arrest after centrosome loss.	Meitinger *et al.*, 2016
HEK293T	Centrinone BioID	The interactions of centriolar satellite proteins are not affected by centrosome loss.	Gheiratmand *et al.*, 2019
RPE-1	Centrinone	Centrosomes indirectly regulate k-fiber plus-ends via spindle length-dependent accumulation of HURP.	Dudka *et al.*, 2019
A549, DU145, GI-1, HCT116, HeLa, MCF-7, PANC-1, SKOV-3, U2OS	Centrinone	NuMA-mediated pathways promote spindle bipolarity independently of centrosomes.	Chinen *et al.*, 2020

## References

[B1] Basto, R., Lau, J., Vinogradova, T., Gardiol, A., Woods, C.G., Khodjakov, A., and Raff, J.W. 2006. Flies without Centrioles. Cell, 125: 1375–1386. 16814722 10.1016/j.cell.2006.05.025

[B2] Beaudouin, J., Gerlich, D., Daigle, N., Eils, R., and Ellenberg, J. 2002. Nuclear Envelope Breakdown Proceeds by Microtubule-Induced Tearing of the Lamina. Cell, 108: 83–96. 11792323 10.1016/s0092-8674(01)00627-4

[B3] Bertran, M.T., Sdelci, S., Regué, L., Avruch, J., Caelles, C., and Roig, J. 2011. Nek9 is a Plk1-activated kinase that controls early centrosome separation through Nek6/7 and Eg5. EMBO J., doi: 10.1038/emboj.2011.179. 10.1038/emboj.2011.179 PMC3155310 21642957

[B4] Bettencourt-Dias, M., Rodrigues-Martins, A., Carpenter, L., Riparbelli, M., Lehmann, L., Gatt, M.K., Carmo, N., Balloux, F., Callaini, G., and Glover, D.M. 2005. SAK/PLK4 Is Required for Centriole Duplication and Flagella Development. Curr. Biol., 15: 2199–2207. 16326102 10.1016/j.cub.2005.11.042

[B5] Bonaccorsi, S., Giansanti, M.G., and Gatti, M. 2000. Spindle assembly in Drosophila neuroblasts and ganglion mother cells. Nat. Cell Biol., 2: 54–56. 10620808 10.1038/71378

[B6] Calarco-Gillam, P.D., Siebert, M.C., Hubble, R., Mitchison, T., and Kirschner, M. 1983. Centrosome development in early mouse embryos as defined by an autoantibody against pericentriolar material. Cell, 35: 621–629. 6652679 10.1016/0092-8674(83)90094-6

[B7] Carazo-Salas, R.E., Guarguaglini, G., Gruss, O.J., Segref, A., Karsenti, E., and Mattaj, I.W. 1999. Generation of GTP-bound Ran by RCC1 is required for chromatin-induced mitotic spindle formation. Nature, 400: 178–181. 10408446 10.1038/22133

[B8] Chabin-Brion, K., Marceiller, J., Perez, F., Settegrana, C., Drechou, A., Durand, G., and Poüs, C. 2001. The Golgi Complex Is a Microtubule-organizing Organelle. Mol. Biol. Cell, 12: 2047–2060. 11452002 10.1091/mbc.12.7.2047 PMC55652

[B9] Chinen, T., Yamamoto, S., Takeda, Y., Watanabe, K., Kuroki, K., Hashimoto, K., Takao, D., and Kitagawa, D. 2020. NuMA assemblies organize microtubule asters to establish spindle bipolarity in acentrosomal human cells. EMBO J., 39: e102378 31782546 10.15252/embj.2019102378 PMC6960446

[B10] Cizmecioglu, O., Arnold, M., Bahtz, R., Settele, F., Ehret, L., Haselmann-Weiß, U., Antony, C., and Hoffmann, I. 2010. Cep152 acts as a scaffold for recruitment of Plk4 and CPAP to the centrosome. J. Cell Biol., 191: 731–739. 21059844 10.1083/jcb.201007107 PMC2983070

[B11] Conduit, P.T., Richens, J.H., Wainman, A., Holder, J., Vicente, C.C., Pratt, M.B., Dix, C.I., Novak, Z.A., Dobbie, I.M., Schermelleh, L., and Raff, J.W. 2014. A molecular mechanism of mitotic centrosome assembly in Drosophila. Elife, 3: 1–23. 10.7554/eLife.03399 PMC4175739 25149451

[B12] Conduit, P.T., Wainman, A., and Raff, J.W. 2015. Centrosome function and assembly in animal cells. Nat. Rev. Mol. Cell Biol., 16: 611–624. 26373263 10.1038/nrm4062

[B13] Cross, M.K. and Powers, M.A. 2009. Learning about cancer from frogs: analysis of mitotic spindles in Xenopus egg extracts. Dis. Model. Mech., 2: 541–547. 19892884 10.1242/dmm.002022 PMC2773725

[B14] Dammermann, A., Müller-Reichert, T., Pelletier, L., Habermann, B., Desai, A., and Oegema, K. 2004. Centriole Assembly Requires Both Centriolar and Pericentriolar Material Proteins. Dev. Cell, 7: 815–829. 15572125 10.1016/j.devcel.2004.10.015

[B15] Du, Q., Taylor, L., Compton, D.A., and Macara, I.G. 2002. LGN Blocks the Ability of NuMA to Bind and Stabilize Microtubules. Curr. Biol., 12: 1928–1933. 12445386 10.1016/s0960-9822(02)01298-8

[B16] Du, Q. and Macara, I.G. 2004. Mammalian Pins Is a Conformational Switch that Links NuMA to Heterotrimeric G Proteins. Cell, 119: 503–516. 15537540 10.1016/j.cell.2004.10.028

[B17] Dudka, D., Castrogiovanni, C., Liaudet, N., Vassal, H., and Meraldi, P. 2019. Spindle-Length-Dependent HURP Localization Allows Centrosomes to Control Kinetochore-Fiber Plus-End Dynamics. Curr. Biol., 29: 3563–3578.e6. 31668617 10.1016/j.cub.2019.08.061

[B18] Dzhindzhev, N.S., Yu, Q.D., Weiskopf, K., Tzolovsky, G., Cunha-Ferreira, I., Riparbelli, M., Rodrigues-Martins, A., Bettencourt-Dias, M., Callaini, G., and Glover, D.M. 2010. Asterless is a scaffold for the onset of centriole assembly. Nature, 467: 714–718. 20852615 10.1038/nature09445

[B19] Efimov, A., Kharitonov, A., Efimova, N., Loncarek, J., Miller, P.M., Andreyeva, N., Gleeson, P., Galjart, N., Maia, A.R.R., McLeod, I.X., Yates, J.R., Maiato, H., Khodjakov, A., Akhmanova, A., and Kaverina, I. 2007. Asymmetric CLASP-Dependent Nucleation of Noncentrosomal Microtubules at the trans-Golgi Network. Dev. Cell, 12: 917–930. 17543864 10.1016/j.devcel.2007.04.002 PMC2705290

[B20] Fong, C.S., Mazo, G., Das, T., Goodman, J., Kim, M., O’Rourke, B.P., Izquierdo, D., and Tsou, M.-F.B. 2016. 53BP1 and USP28 mediate p53-dependent cell cycle arrest in response to centrosome loss and prolonged mitosis. Elife, 5: e16270. 27371829 10.7554/eLife.16270 PMC4946878

[B21] Fu, J., Lipinszki, Z., Rangone, H., Min, M., Mykura, C., Chao-Chu, J., Schneider, S., Dzhindzhev, N.S., Gottardo, M., Riparbelli, M.G., Callaini, G., and Glover, D.M. 2016. Conserved molecular interactions in centriole-to-centrosome conversion. Nat. Cell Biol., 18: 87–99. 26595382 10.1038/ncb3274 PMC4719191

[B22] Gaglio, T., Saredi, A., and Compton, D.A. 1995. NuMA is required for the organization of microtubules into aster-like mitotic arrays. J. Cell Biol., 131: 693–708 7593190 10.1083/jcb.131.3.693 PMC2120610

[B23] Gheiratmand, L., Coyaud, E., Gupta, G.D., Laurent, E.M., Hasegan, M., Prosser, S.L., Gonçalves, J., Raught, B., and Pelletier, L. 2019. Spatial and proteomic profiling reveals centrosome‐independent features of centriolar satellites. EMBO J., 38: 1–22. 10.15252/embj.2018101109 PMC6627244 31304627

[B24] Groen, A.C., Needleman, D., Brangwynne, C., Gradinaru, C., Fowler, B., Mazitschek, R., and Mitchison, T.J. 2008. A novel small-molecule inhibitor reveals a possible role of kinesin-5 in anastral spindle-pole assembly. J. Cell Sci., 121: 2293–2300. 18559893 10.1242/jcs.024018 PMC2902979

[B25] Guizzunti, G. and Seemann, J. 2016. Mitotic Golgi disassembly is required for bipolar spindle formation and mitotic progression. Proc. Natl. Acad. Sci., 113: E6590–E6599. 27791030 10.1073/pnas.1610844113 PMC5087019

[B26] Habedanck, R., Stierhof, Y.-D., Wilkinson, C.J., and Nigg, E.A. 2005. The Polo kinase Plk4 functions in centriole duplication. Nat. Cell Biol., 7: 1140–1146. 16244668 10.1038/ncb1320

[B27] Haren, L. and Merdes, A. 2002. Direct binding of NuMA to tubulin is mediated by a novel sequence motif in the tail domain that bundles and stabilizes microtubules. J. Cell Sci., 115: 1815–24. 11956313 10.1242/jcs.115.9.1815

[B28] Harrington, E.A., Bebbington, D., Moore, J., Rasmussen, R.K., Ajose-Adeogun, A.O., Nakayama, T., Graham, J.A., Demur, C., Hercend, T., Diu-Hercend, A., Su, M., Golec, J.M.C., and Miller, K.M. 2004. VX-680, a potent and selective small-molecule inhibitor of the Aurora kinases, suppresses tumor growth in vivo. Nat. Med., 10: 262–267. 14981513 10.1038/nm1003

[B29] Hata, S., Pastor Peidro, A., Panic, M., Liu, P., Atorino, E., Funaya, C., Jäkle, U., Pereira, G., and Schiebel, E. 2019. The balance between KIFC3 and EG5 tetrameric kinesins controls the onset of mitotic spindle assembly. Nat. Cell Biol., 21: 1138–1151. 31481795 10.1038/s41556-019-0382-6

[B30] Hatch, E.M., Kulukian, A., Holland, A.J., Cleveland, D.W., and Stearns, T. 2010. Cep152 interacts with Plk4 and is required for centriole duplication. J. Cell Biol., 191: 721–729. 21059850 10.1083/jcb.201006049 PMC2983069

[B31] Heald, R., Tournebize, R., Blank, T., Sandaltzopoulos, R., Becker, P., Hyman, A., and Karsenti, E. 1996. Self-organization of microtubules into bipolar spindles around artificial chromosomes in Xenopus egg extracts. Nature, 382: 420–425. 8684481 10.1038/382420a0

[B32] Henriques, A.C., Ribeiro, D., Pedrosa, J., Sarmento, B., Silva, P.M.A., and Bousbaa, H. 2019. Mitosis inhibitors in anticancer therapy: When blocking the exit becomes a solution. Cancer Lett., 440–441: 64–81. 10.1016/j.canlet.2018.10.005 30312726

[B33] Holubcová, Z., Blayney, M., Elder, K., and Schuh, M. 2015. Error-Prone Chromosome-Mediated Spindle Assembly Favors Chromosome Segregation Defects in Human Oocytes. Science, 348: 1143–1147. 26045437 10.1126/science.aaa9529 PMC4477045

[B34] Hornick, J.E., Mader, C.C., Tribble, E.K., Bagne, C.C., Vaughan, K.T., Shaw, S.L., and Hinchcliffe, E.H. 2011. Amphiastral Mitotic Spindle Assembly in Vertebrate Cells Lacking Centrosomes. Curr. Biol., 21: 598–605. 21439826 10.1016/j.cub.2011.02.049 PMC3075362

[B35] Izquierdo, D., Wang, W.-J., Uryu, K., and Tsou, M.-F.B. 2014. Stabilization of Cartwheel-less Centrioles for Duplication Requires CEP295-Mediated Centriole-to-Centrosome Conversion. Cell Rep., 8: 957–965. 25131205 10.1016/j.celrep.2014.07.022 PMC4152953

[B36] Kalab, P., Pu, R.T., and Dasso, M. 1999. The Ran GTPase regulates mitotic spindle assembly. Curr. Biol., 9: 481–484. 10322113 10.1016/s0960-9822(99)80213-9

[B37] Kapitein, L.C., Peterman, E.J.G., Kwok, B.H., Kim, J.H., Kapoor, T.M., and Schmidt, C.F. 2005. The bipolar mitotic kinesin Eg5 moves on both microtubules that it crosslinks. Nature, 435: 114–118. 15875026 10.1038/nature03503

[B38] Karsenti, E., Newport, J., Hubble, R., and Kirschner, M. 1984. Interconversion of metaphase and interphase microtubule arrays, as studied by the injection of centrosomes and nuclei into Xenopus eggs. J. Cell Biol., 98: 1730–1745. 6725396 10.1083/jcb.98.5.1730 PMC2113192

[B39] Kaseda, K., McAinsh, A.D., and Cross, R.A. 2012. Dual pathway spindle assembly increases both the speed and the fidelity of mitosis. Biol. Open, 1: 12–18. 23213363 10.1242/bio.2011012 PMC3507165

[B40] Khodjakov, A., Cole, R.W., Oakley, B.R., and Rieder, C.L. 2000. Centrosome-independent mitotic spindle formation in vertebrates. Curr. Biol., 10: 59–67. 10662665 10.1016/s0960-9822(99)00276-6

[B41] Kleylein-Sohn, J., Westendorf, J., Le Clech, M., Habedanck, R., Stierhof, Y.-D., and Nigg, E.A. 2007. Plk4-Induced Centriole Biogenesis in Human Cells. Dev. Cell, 13: 190–202. 17681131 10.1016/j.devcel.2007.07.002

[B42] Lambrus, B.G., Daggubati, V., Uetake, Y., Scott, P.M., Clutario, K.M., Sluder, G., and Holland, A.J. 2016. A USP28–53BP1–p53–p21 signaling axis arrests growth after centrosome loss or prolonged mitosis. J. Cell Biol., 214: 143–153. 27432896 10.1083/jcb.201604054 PMC4949452

[B43] Leidel, S., Delattre, M., Cerutti, L., Baumer, K., and Gönczy, P. 2005. SAS-6 defines a protein family required for centrosome duplication in C. elegans and in human cells. Nat. Cell Biol., 7: 115–125. 15665853 10.1038/ncb1220

[B44] Lin, Y.-C., Chang, C.-W., Hsu, W.-B., Tang, C.-J.C., Lin, Y.-N., Chou, E.-J., Wu, C.-T., and Tang, T.K. 2013. Human microcephaly protein CEP135 binds to hSAS-6 and CPAP, and is required for centriole assembly. EMBO J., 32: 1141–1154. 23511974 10.1038/emboj.2013.56 PMC3630357

[B45] Loughlin, R., Heald, R., and Nédélec, F. 2010. A computational model predicts Xenopus meiotic spindle organization. J. Cell Biol., 191: 1239–1249. 21173114 10.1083/jcb.201006076 PMC3010074

[B46] Matthies, H.J.G., McDonald, H.B., Goldstein, L.S.B., and Theurkauf, W.E. 1996. Anastral meiotic spindle morphogenesis: role of the non-claret disjunctional kinesin-like protein. J. Cell Biol., 134: 455–464. 8707829 10.1083/jcb.134.2.455 PMC2120873

[B47] Meitinger, F., Anzola, J.V., Kaulich, M., Richardson, A., Stender, J.D., Benner, C., Glass, C.K., Dowdy, S.F., Desai, A., Shiau, A.K., and Oegema, K. 2016. 53BP1 and USP28 mediate p53 activation and G1 arrest after centrosome loss or extended mitotic duration. J. Cell Biol., 214: 155–166. 27432897 10.1083/jcb.201604081 PMC4949453

[B48] Merdes, A., Ramyar, K., Vechio, J.D., and Cleveland, D.W. 1996. A Complex of NuMA and Cytoplasmic Dynein Is Essential for Mitotic Spindle Assembly. Cell, 87: 447–458. 8898198 10.1016/s0092-8674(00)81365-3

[B49] Merdes, A., Heald, R., Samejima, K., Earnshaw, W.C., and Cleveland, D.W. 2000. Formation of spindle poles by dynein/dynactin-dependent transport of NuMA. J. Cell Biol., 149: 851–862. 10811826 10.1083/jcb.149.4.851 PMC2174573

[B50] Natsume, T., Kiyomitsu, T., Saga, Y., and Kanemaki, M.T. 2016. Rapid Protein Depletion in Human Cells by Auxin-Inducible Degron Tagging with Short Homology Donors. Cell Rep., 15: 210–218. 27052166 10.1016/j.celrep.2016.03.001

[B51] Nigg, E.A. and Raff, J.W. 2009. Centrioles, Centrosomes, and Cilia in Health and Disease. Cell, 139: 663–678. 19914163 10.1016/j.cell.2009.10.036

[B52] Nigg, E.A. and Holland, A.J. 2018. Once and only once: mechanisms of centriole duplication and their deregulation in disease. Nat. Rev. Mol. Cell Biol., 19: 297–312. 29363672 10.1038/nrm.2017.127 PMC5969912

[B53] Nishimura, K., Fukagawa, T., Takisawa, H., Kakimoto, T., and Kanemaki, M. 2009. An auxin-based degron system for the rapid depletion of proteins in nonplant cells. Nat. Methods, 6: 917–922. 19915560 10.1038/nmeth.1401

[B54] Ohta, M., Ashikawa, T., Nozaki, Y., Kozuka-Hata, H., Goto, H., Inagaki, M., Oyama, M., and Kitagawa, D. 2014. Direct interaction of Plk4 with STIL ensures formation of a single procentriole per parental centriole. Nat. Commun., 5: 5267. 25342035 10.1038/ncomms6267 PMC4220463

[B55] Okumura, M., Natsume, T., Kanemaki, M.T., and Kiyomitsu, T. 2018. Dynein–Dynactin–NuMA clusters generate cortical spindle-pulling forces as a multi-arm ensemble. Elife, 7: e36559. 29848445 10.7554/eLife.36559 PMC6037482

[B56] Paweletz, N., Mazia, D., and Finze, E.-M. 1984. The centrosome cycle in the mitotic cycle of sea urchin eggs. Exp. Cell Res., 152: 47–65. 6538848 10.1016/0014-4827(84)90229-5

[B57] Petry, S., Pugieux, C., Nedelec, F.J., and Vale, R.D. 2011. Augmin promotes meiotic spindle formation and bipolarity in Xenopus egg extracts. Proc. Natl. Acad. Sci., 108: 14473–14478. 21844347 10.1073/pnas.1110412108 PMC3167534

[B58] Prosser, S.L. and Pelletier, L. 2017. Mitotic spindle assembly in animal cells: a fine balancing act. Nat. Rev. Mol. Cell Biol., 18: 187–201. 28174430 10.1038/nrm.2016.162

[B59] Quarantotti, V., Chen, J., Tischer, J., Gonzalez Tejedo, C., Papachristou, E.K., D’Santos, C.S., Kilmartin, J.V, Miller, M.L., and Gergely, F. 2019. Centriolar satellites are acentriolar assemblies of centrosomal proteins. EMBO J., 38: 1–18. 10.15252/embj.2018101082 PMC6627235 31304626

[B60] Raaijmakers, J.A., Van Heesbeen, R.G.H.P., Meaders, J.L., Geers, E.F., Fernandez-Garcia, B., Medema, R.H., and Tanenbaum, M.E. 2012. Nuclear envelope-associated dynein drives prophase centrosome separation and enables Eg5-independent bipolar spindle formation. EMBO J., 31: 4179–4190. 23034402 10.1038/emboj.2012.272 PMC3492733

[B61] Rattner, J.B. and Phillips, S.G. 1973. INDEPENDENCE OF CENTRIOLE FORMATION AND DNA SYNTHESIS. J. Cell Biol., 57: 359–372. 4696548 10.1083/jcb.57.2.359 PMC2108974

[B62] Rattner, J.B. and Berns, M.W. 1976. Centriole behavior in early mitosis of rat kangaroo cells (PTK2). Chromosoma, 54: 387–395. 1253644 10.1007/BF00292817

[B63] Rivero, S., Cardenas, J., Bornens, M., and Rios, R.M. 2009. Microtubule nucleation at the cis-side of the Golgi apparatus requires AKAP450 and GM130. EMBO J., 28: 1016–1028. 19242490 10.1038/emboj.2009.47 PMC2683699

[B64] Ríos, R.M., Sanchís, A., Tassin, A.M., Fedriani, C., and Bornens, M. 2004. GMAP-210 Recruits γ-Tubulin Complexes to cis-Golgi Membranes and Is Required for Golgi Ribbon Formation. Cell, 118: 323–335. 15294158 10.1016/j.cell.2004.07.012

[B65] Roux, K.J., Kim, D.I., Raida, M., and Burke, B. 2012. A promiscuous biotin ligase fusion protein identifies proximal and interacting proteins in mammalian cells. J. Cell Biol., 196: 801–810. 22412018 10.1083/jcb.201112098 PMC3308701

[B66] Schuh, M. and Ellenberg, J. 2007. Self-Organization of MTOCs Replaces Centrosome Function during Acentrosomal Spindle Assembly in Live Mouse Oocytes. Cell, 130: 484–498. 17693257 10.1016/j.cell.2007.06.025

[B67] Sir, J.-H., Pütz, M., Daly, O., Morrison, C.G., Dunning, M., Kilmartin, J.V., and Gergely, F. 2013. Loss of centrioles causes chromosomal instability in vertebrate somatic cells. J. Cell Biol., 203: 747–756. 24297747 10.1083/jcb.201309038 PMC3857480

[B68] Sloane, D.A., Trikic, M.Z., Chu, M.L.H., Lamers, M.B.A.C., Mason, C.S., Mueller, I., Savory, W.J., Williams, D.H., and Eyers, P.A. 2010. Drug-Resistant Aurora A Mutants for Cellular Target Validation of the Small Molecule Kinase Inhibitors MLN8054 and MLN8237. ACS Chem. Biol., 5: 563–576. 20426425 10.1021/cb100053q

[B69] Smith, E., Hégarat, N., Vesely, C., Roseboom, I., Larch, C., Streicher, H., Straatman, K., Flynn, H., Skehel, M., Hirota, T., Kuriyama, R., and Hochegger, H. 2011. Differential control of Eg5-dependent centrosome separation by Plk1 and Cdk1. EMBO J., 30: 2233–2245. 21522128 10.1038/emboj.2011.120 PMC3117641

[B70] Stevens, N.R., Dobbelaere, J., Brunk, K., Franz, A., and Raff, J.W. 2010. Drosophila Ana2 is a conserved centriole duplication factor. J. Cell Biol., 188: 313–323. 20123993 10.1083/jcb.200910016 PMC2819680

[B71] Tang, C.J.C., Lin, S.Y., Hsu, W. Bin, Lin, Y.N., Wu, C.T., Lin, Y.C., Chang, C.W., Wu, K.S., and Tang, T.K. 2011. The human microcephaly protein STIL interacts with CPAP and is required for procentriole formation. EMBO J., 30: 4790–4804. 22020124 10.1038/emboj.2011.378 PMC3243611

[B72] Tischer, J. and Gergely, F. 2019. Anti-mitotic therapies in cancer. J. Cell Biol., 218: 10–11. 30545842 10.1083/jcb.201808077 PMC6314557

[B73] Toso, A., Winter, J.R., Garrod, A.J., Amaro, A.C., Meraldi, P., and McAinsh, A.D. 2009. Kinetochore-generated pushing forces separate centrosomes during bipolar spindle assembly. J. Cell Biol., 184: 365–372. 19204145 10.1083/jcb.200809055 PMC2646558

[B74] Tsou, M.-F.B. and Stearns, T. 2006. Mechanism limiting centrosome duplication to once per cell cycle. Nature, 442: 947–951. 16862117 10.1038/nature04985

[B75] Tsou, M.-F.B., Wang, W.-J., George, K.A., Uryu, K., Stearns, T., and Jallepalli, P.V. 2009. Polo Kinase and Separase Regulate the Mitotic Licensing of Centriole Duplication in Human Cells. Dev. Cell, 17: 344–354. 19758559 10.1016/j.devcel.2009.07.015 PMC2746921

[B76] Tsuchiya, Y., Yoshiba, S., Gupta, A., Watanabe, K., and Kitagawa, D. 2016. Cep295 is a conserved scaffold protein required for generation of a bona fide mother centriole. Nat. Commun., 7: 12567. 27562453 10.1038/ncomms12567 PMC5007451

[B77] Vidwans, S.J., Wong, M.L., and O’Farrell, P.H. 1999. Mitotic Regulators Govern Progress through Steps in the Centrosome Duplication Cycle. J. Cell Biol., 147: 1371–1378. 10613895 10.1083/jcb.147.7.1371 PMC2174235

[B78] Waters, J., Cole, R., and Rieder, C. 1993. The force-producing mechanism for centrosome separation during spindle formation in vertebrates is intrinsic to each aster. J. Cell Biol., 122: 361–372. 8320259 10.1083/jcb.122.2.361 PMC2119639

[B79] Werner, S., Pimenta-Marques, A., and Bettencourt-Dias, M. 2017. Maintaining centrosomes and cilia. J. Cell Sci., 130: 3789–3800. 29142065 10.1242/jcs.203505

[B80] Whitehead, C.M. and Rattner, J.B. 1998. Expanding the role of HsEg5 within the mitotic and post-mitotic phases of the cell cycle. J. Cell Sci., 111: 2551–2561. 9701554 10.1242/jcs.111.17.2551

[B81] Wilde, A. 1999. Stimulation of Microtubule Aster Formation and Spindle Assembly by the Small GTPase Ran. Science, 284: 1359–1362. 10334991 10.1126/science.284.5418.1359

[B82] Wong, Y.L., Anzola, J.V., Davis, R.L., Yoon, M., Motamedi, A., Kroll, A., Seo, C.P., Hsia, J.E., Kim, S.K., Mitchell, J.W., Mitchell, B.J., Desai, A., Gahman, T.C., Shiau, A.K., and Oegema, K. 2015. Reversible centriole depletion with an inhibitor of Polo-like kinase 4. Science, 348: 1155–1160. 25931445 10.1126/science.aaa5111 PMC4764081

[B83] Wu, J., de Heus, C., Liu, Q., Bouchet, B.P., Noordstra, I., Jiang, K., Hua, S., Martin, M., Yang, C., Grigoriev, I., Katrukha, E.A., Altelaar, A.F.M., Hoogenraad, C.C., Qi, R.Z., Klumperman, J., and Akhmanova, A. 2016. Molecular Pathway of Microtubule Organization at the Golgi Apparatus. Dev. Cell, 39: 44–60. 27666745 10.1016/j.devcel.2016.08.009

[B84] Zhang, C., Hughes, M., and Clarke, P.R. 1999. Ran-GTP stabilises microtubule asters and inhibits nuclear assembly in Xenopus egg extracts. J. Cell Sci., 112: 2453–2461. 10381400 10.1242/jcs.112.14.2453

